# Machine learning for exploring neurophysiological functionality in multiple sclerosis based on trigeminal and hand blink reflexes

**DOI:** 10.1038/s41598-022-24720-6

**Published:** 2022-12-06

**Authors:** Monica Biggio, Daniele Caligiore, Federico D’Antoni, Marco Bove, Mario Merone

**Affiliations:** 1 Department of Experimental Medicine, Section of Human Physiology and Centro Polifunzionale di Scienze Motorie, University of Genoa, Viale Benedetto XV 3, 16132 Genoa, Italy; 2 Computational and Translational Neuroscience Laboratory, Institute of Cognitive Sciences and Technologies, National Research Council (CTNLab-ISTC-CNR), Via San Martino della Battaglia 44, 00185 Rome, Italy; 3 AI2Life s.r.l., Innovative Start-Up, ISTC-CNR Spin-Off, Via Sebino 32, 00199 Rome, Italy; 4 Research Unit of Computer Systems and Bioinformatics, Department of Engineering, Università Campus Bio-Medico di Roma, Via Alvaro del Portillo, 21, 00141 Rome, Italy; 5 IRCCS Ospedale Policlinico San Martino, Largo Rosanna Benzi, 10, 16132 Genoa, Italy

**Keywords:** Multiple sclerosis, Diseases of the nervous system

## Abstract

Brainstem dysfunctions are very common in Multiple Sclerosis (MS) and are a critical predictive factor for future disability. Brainstem functionality can be explored with blink reflexes, subcortical responses consisting in a blink following a peripheral stimulation. Some reflexes are already employed in clinical practice, such as Trigeminal Blink Reflex (TBR). Here we propose for the first time in MS the exploration of Hand Blink Reflex (HBR), which size is modulated by the proximity of the stimulated hand to the face, reflecting the extension of the peripersonal space. The aim of this work is to test whether Machine Learning (ML) techniques could be used in combination with neurophysiological measurements such as TBR and HBR to improve their clinical information and potentially favour the early detection of brainstem dysfunctionality. HBR and TBR were recorded from a group of People with MS (PwMS) with Relapsing-Remitting form and from a healthy control group. Two AdaBoost classifiers were trained with TBR and HBR features each, for a binary classification task between PwMS and Controls. Both classifiers were able to identify PwMS with an accuracy comparable and even higher than clinicians. Our results indicate that ML techniques could represent a tool for clinicians for investigating brainstem functionality in MS. Also, HBR could be promising when applied in clinical practice, providing additional information about the integrity of brainstem circuits potentially favouring early diagnosis.

## Introduction

Multiple sclerosis (MS) is an acquired inflammatory and demyelinating neurodegenerative disease. It affects the central nervous system (CNS), producing a loss of motor and sensory function. MS is one of the most common causes of neurological disability in young adults and has a great functionally and financially impact on quality of life^1^. The prevalence of this disorder ranges from 50 to 300 per 100,000, with approximately 2.3 million people affected worldwide^2^.

Blink Reflex (BR) alterations are the manifestations of brainstem dysfunctions, that are known to be common in People with MS (PwMS)^3^. BR is a prototypical defensive reflex that can be elicited by abrupt and intense stimuli in various sensory modalities: visual, auditory and somatosensory^4^. BR could show a great diagnostic potential^5,6^, supporting the diagnosis and follow-up of patients with relapsing-remitting MS^7^. Several brainstem reflexes show distinctive alterations in MS, reflecting areas of brainstem damage^8,9^. A well known BR that shows characteristic alteration in MS is the Trigeminal Blink Reflex (TBR), elicited by the electrical stimulation of the supraorbital nerve. The TBR consists of a short-latency, ipsilateral component (R1), followed by a second bilateral component (R2). Other pathologies, such as trigeminal neuralgia (TN), showed the importance of relying on TBR in diagnostic and characterization of patients pathology^10^. For example, discrimination between idiopathic or classical TN is improved based on BR characteristics^11^. TBR alterations are critical to find clinically isolated syndrome (CIS) in Multiple Sclerosis, but its specific components alterations could still be tricky to interpret^6^. For example, Mikropoulos and colleagues found that the presence of brainstem lesions does not significantly affect TBR sensitivity, and their results underscored the influence of supratentorial MS lesions on the TBR response^12^. Degirmenci and colleagues, on the contrary, found a positive correlation between brainstem lesions and contralateral R2 latencies but proposed that brainstem lesions are possibly not the only ones responsible for TBR alteration in MS^13^. Battery combining multiple brainstem reflexes showed significantly higher sensitivity in MS assessment than clinical and Magnetic Resonance Imaging (MRI) procedures taken together^8^. For this reason, it is fundamental to explore different subcortical reflexes to study the different patterns of alteration in PwMS.

In this paper, we investigate another BR that is never been explored before in MS: the Hand Blink Reflex (HBR). HBR is a subcortical response elicited by the electrical stimulation of the median nerve at the wrist and recorded from the orbicularis oculi muscles. It is characterized by a bilateral R2 component similar to TBR. The main characteristic of HBR is that the proximity of the stimulated hand to the face modulates this reflex. In particular, the size of the reflex increases when the hand is inside the defensive peripersonal space (DPPS) of the face^14–17^. Since the novelty of HBR exploration in MS, we hypothesize that it could be introduced in clinical practice in MS to explore brainstem functionality.

The goal of this work is to test whether Machine Learning (ML) techniques could be combined with neurophysiological approaches based on TBR and HBR to improve their clinical information and favour early MS detection. ML is increasingly used to improve image analysis and efficacy of care in MS^18^. It has been also applied with success on patients reported outcomes and clinical-assessed outcomes in order to predict the evolution of the pathology^19^. To our knowledge, ML has never been applied to the study of BR, specifically in the field of MS diagnosis. Here, we tested whether an ML algorithm can distinguish between patients and healthy subjects based on TBR or HBR features. We verified whether HBR could be impaired in PwMS as other brainstem reflexes, showing different pattern of impairment with respect to the well-known TBR. To reach this goal we analyzed two datasets of TBR and HBR data we collected over a Relapsing Remitting group of PwMS and a control group of healthy age - matching subjects. We developed two Adaptive Boosting (AdaBoost) classifiers^20,21^ trained with TBR and HBR features each, to distinguish between PwMS and a control group. This is the first time that different brainstem reflexes are taken into consideration and compared with innovative ML methods. Since PwMS are characterized by a wide variety of manifestations, accompanying clinical examination with ML could help take account of the multiple patterns of alteration of BR response.

## Methods

The study was conducted in accordance with the 2013 revision of the Declaration of Helsinki on human experimentation, and it was approved by the local ethics committee (prot. $\hbox {n}^\circ$ 452REG2015 - 107-17/12/18, Comitato Etico Regionale Liguria, IRCCS Azienda Ospedaliera Universitaria San Martino-IST, Genoa, Italy). Subjects participated in this study after giving their written informed consent.

### Participants

With the aim to investigate HBR and TBR responses, two groups underwent sessions of non-invasive electromyography: a group of 17 people with MS (13 F, 4 M; age 51.71 ± 7.97 years) with Relapsing-Remitting form EDSS < 4) and 16 age-matched healthy controls (10 F, 6 M; age 48.8 ± 9.5 years). The PwMS were selected with a diagnosis of definite relapsing-remitting MS according to revised 2010 McDonald criteria^22^, considering the following as inclusion criteria: age of more than or equal to 18 years; EDSS score less than or equal to 4, being relapse-free or stable in the last three months. Exclusion criteria were: a score lower than or equal to 24 at the Mini-Mental State Examination^23^ to exclude persons with severe cognitive impairment; presence of sensitivity impairments on the basis of EDSS sensory function subscale score to exclude ; presence of additional neurological or psychiatric disease; history of epilepsy, seizures, febrile seizures, head trauma, stroke, drug or alcohol abuse; use of medications influencing cerebellar function and/or muscle tone, e.g. anti-epileptic drugs, benzodiazepine, antidepressants, B-blockers; inability to give informed consent.

### Experimental setup

Reflex responses were elicited using a surface bipolar electrode connected to a constant current stimulator (DS7AH HV, Digitimer). As the stimulator provided constant current pulses, the trial-to-trial variability of the stimulation intensity was negligible.

The TBR response was elicited by administering percutaneous electrical stimulation of the supraorbital branch of the trigeminal nerve (supraorbital nerve, SON). Stimulus intensity was adjusted to elicit in each participant clear TBR responses, with the stimulus set to 200% of the patient’s sensitivity threshold (mean stimulus intensities were 6.13mA ± 1.60 ). The stimulus pulse duration was 200 $\mu$s.

The HBR response was elicited by administering transcutaneous electrical stimuli to the median nerve at the right wrist. Stimulus intensity was adjusted to elicit in each participant clear HBR responses (mean stimulus intensities were 40.06mA ± 23.72). None of the participants reported painful sensations elicited by the stimulation. The stimulus pulse duration was 200 $\upmu \hbox {s}$, and the interstimulus interval was 30 s. A twin-axis electronic goniometer (TSD130B, BIOPAC System) connected to a BIOPAC MP100 system was used to measure and record the elbow angle during movement execution. In Voluntary Movement conditions, this device allowed the automatic delivery of the electrical stimulation when the elbow angle corresponded to one of the three predetermined stimulation positions.

EMG activity of HBR and TBR was recorded by means of two MP100 BIOPAC EMG channels from the orbicularis oculi muscles bilaterally, using two pairs of bipolar surface electrodes with the active electrode over the mid lower eyelid and the reference electrode laterally to the outer canthus. Signals were amplified and digitized at 1 kHz. Ten TBR responses were recorded bilaterally from both side of stimulation, for a total of twenty trials. Ten responses were also recorded bilaterally for each HBR conditions for each side of stimulation, for a total of forty trials. This is the same experimental apparatus used by Bisio^15^ and Mercante^24^.

### Experimental procedure

TBR and HBR were evoked bilaterally and were randomized throughout subjects. For TBR, electrical stimulation were administered 10 times for each side of stimulation. For HBR, electrical stimulation were administered in two target position with respect to the face: when the elbow angle was: $10^\circ$ less than the maximal arm extension (FAR position); $10^\circ$ more than the maximal elbow flexion (NEAR position). In static condition subjects were asked to assume one of the two target positions and the stimulations were administered manually. In voluntary movements conditions subjects were asked to move the elbow from a position of maximum extension, far from the face, towards a position close to the face (Up-movement) or from near the face to a far position (Down-moving). The stimulations were automatically delivered in one of the target positions both Up-moving that Down-moving by a twin-axis electronic goniometer. EMG TBR and HBR signals recorded from each participant were filtered and rectified (band pass 5–5000 Hz). Responses were averaged separately in each condition and for each participant.

### Dataset

Two AdaBoost^20^ classifiers were trained, tested, and validated using different datasets that have the two groups of subjects as targets. A first dataset was created using data recorded from TBR experiment. We considered the area under the curve (AUC, mV x ms), the latency (ms) and the duration (ms) of each TBR average waveform recorded from both eyes and elicited from both forehead side for bilateral (R2)^14^ components, and the latency and the duration of the early ipsilateral component (R1), for a total of 16 features. The second dataset was created using data recorded from HBR experiment. As parameter we considered the area under the curve, the latency, and the duration of each HBR average waveform recorded from both eyes and elicited from both wrists in static or voluntary movement condition^15^ in two target positions with respect to the face (NEAR and FAR), for a total of 72 features. Datasets were subsequently processed in Python (distribution 3.7.1) using Pandas libraries. No missing values were present in the two datasets.

**Figure 1:**
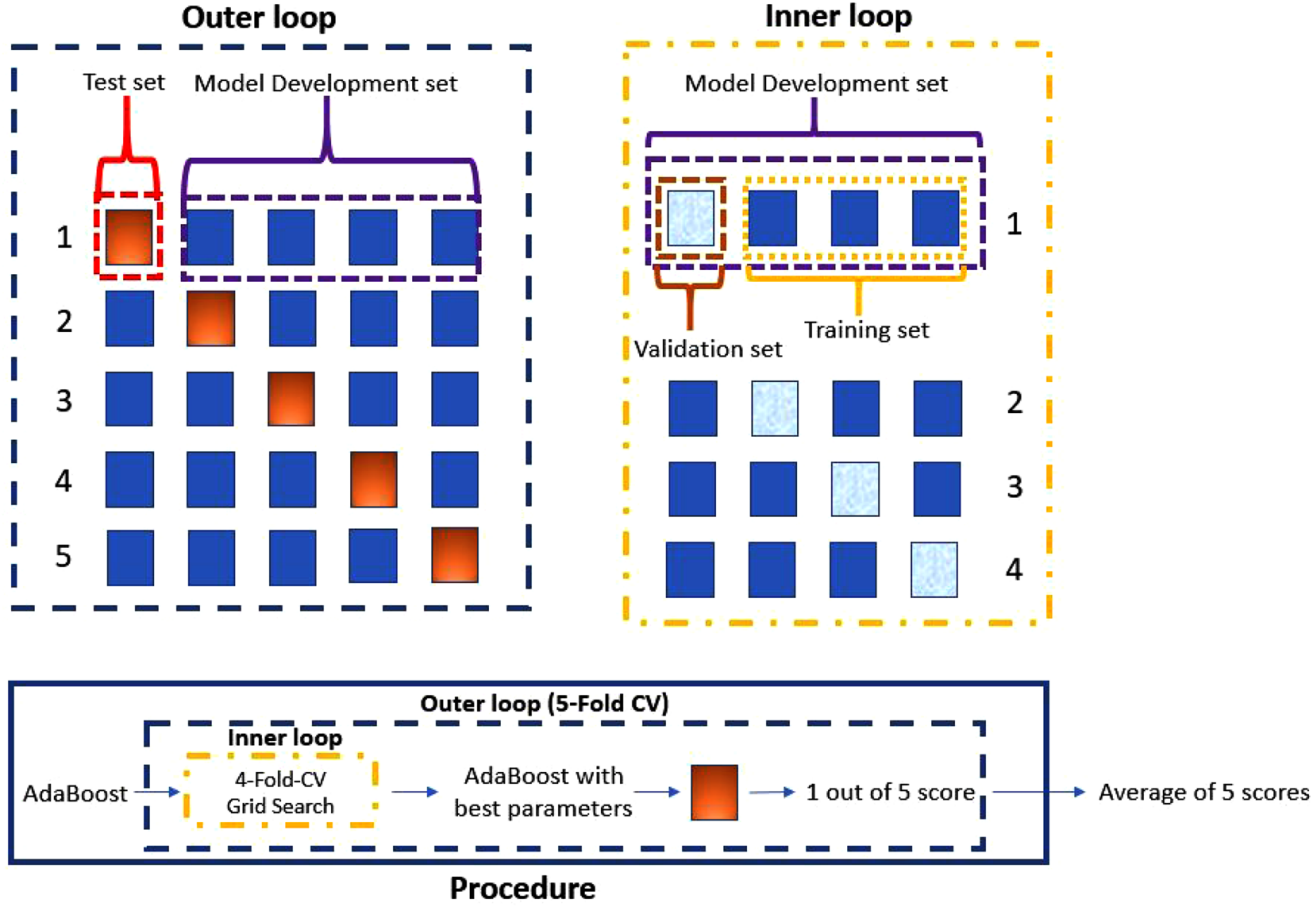
Schematic representation of the Nested-5-Fold-Cross-Validation procedure used in this work.

### Classifier

The features (i.e. TBR or HBR characteristics) are used to train the predictive algorithms for the binary (PwMS/healthy) classification task. During preliminary tests, we investigated different classification models, namely AdaBoost, k-nearest neighbors (k-NN), Support Vector Machine (SVM), Random Forest, and feedforward neural network (NN). For each model, we investigated the most widely used configurations in the literature through the medium of a Nested 5-fold-cross-validation procedure, as described in the next section. With regard to the AdaBoost, we varied the number of estimators between 10 and 70 while considering SAMME and SAMME.R as algorithms, with learning rate values ranging from $10^{-3}$ to 0.7; with regard to the k-NN, we investigated a number of neighbors ranging from 3 to 19 while considering Euclidean and Manhattan distance; with regard to the SVM, we investigated linear, polynomial, sigmoid, and radial basis function kernels while varying the regularization parameter between 0.1 and 100 and the kernel coefficient between $10^{-3}$ and 1; with regard to the RF, the number of estimators varies between 30 and 1000 while the maximum depth varies between 50 and 110, the minimum number of samples per leaf varies between 3 and 5, and the minimum number of samples required per split varies between 8 and 12; finally, the feedforward NN model presents one hidden layer with 32 neurons each having ReLU activation function and one output neuron with sigmoid activation function, whereas we tested different batch sizes ranging from 10 to 100, different maximum number of epochs ranging from 10 to 200, and different optimizers including Stochastic Gradient Descent, RMSprop, Adagrad, Adadelta, Adam, Adamax, and Nadam. We selected AdaBoost as a proposed model due to its better performance and greater interpretability compared to the other models. Moreover, it provides an immediate way to determine which features are most important for the classification task. The core principle of AdaBoost is to fit a sequence of weak learners on repeatedly modified versions of the data. The predictions from all of them are finally combined through a weighted majority vote (or sum) to produce the final prediction. In this work, we use Decision Tree classifiers as weak learners. Data modifications at each so-called boosting iteration consist of applying weights to each of the training samples as follows:

1. The first step trains a weak learner on the original data.
2. At a given step, those training examples that were incorrectly predicted by the boosted model induced at the previous step have their weights increased, whereas the weights are decreased for examples that were predicted correctly.
3. For each successive iteration, the sample weights are individually modified and the learning algorithm is reapplied to the re-weighted data.

In this way, examples that are difficult to predict receive ever-increasing influence as iterations proceed. Each subsequent weak learner is thereby forced to concentrate on the examples that are missed by the previous ones in the sequence^25^.

### Validation Procedure

To train the system and evaluate its performance, we used the Nested-5-Fold-Cross-Validation procedure for all the classifier taken into consideration. With particular regard to the AdaBoost, we used this method to select the optimal number of weak learners, the learning rate and the optimization algorithm, and finally to achieve the average performance of the ensemble^26,27^. In this way, we avoid model overfitting and optimistically-biased estimates of model performance.

This procedure is composed of two Cross Validation (CV) loops, and in detail:

- in the outer CV loop, designed to obtain an unbiased estimate of the model performance, the dataset is partitioned into the ‘Model Development Set’ and the ‘Test Set’ by creating 5 evenly-divided folds. This is schematized in the upper left part of the Fig. 1;
- For each iteration of the outer CV loop, an entire inner CV loop was performed. The inner CV loop was designed to select the optimal hyperparameters for the final model through a Grid Search technique with the accuracy on validation set as selection score^28^. In each inner loop, the ‘Model Development Set’ was further partitioned in 4 evenly-divided folds, obtaining the ‘Training Set’ and the ’Validation Set’. This is schematized in the upper right part of the Fig. 1.

During each inner loop, a grid search was performed to detect the optimal combination of parameters with regards to the number of learners, the learning rate and the optimization algorithm. At the end of each inner loop, a model was trained from scratch on the whole Model Development set using the optimal parameters, which were selected based on the Accuracy achieved on the different Validation sets; finally, the optimized model was tested on the Test set to evaluate unbiased performance. The complete procedure is outlined in the lower part of Fig. 1. It is worth noting that by using 5 folds in the outer loop and 4 folds in the inner loop, each fold consists of 6 examples.

### Metrics performance

As we perform a classification task, we report the results in terms of Accuracy, Recall, Precision and F1-Score. We are considering a binary classification task, e.g., Positive vs Negative. Given a test set composed of *N* samples, defined the True Positives *TP* as the number of Positive samples correctly classified, and the True Negatives *TN* as the number of Negative samples correctly classified, Accuracy is defines as:

1 $$\begin{aligned} Acc \% = \frac{TP+TN}{N} \times 100 \end{aligned}$$

thus, greater values correspond to better performance. In practice, Accuracy represents the amount of samples correctly classified^29^. Recall and Precision can be computed separately for each class. Defined the False Positives *FP* and False Negatives *FN* as the number of misclassified Negative/Positive samples, Recall and Precision for each class are defined as:

2 $$\begin{aligned} Recall = \frac{TP}{TP+FN} \qquad Precision = \frac{TP}{TP+FP} \end{aligned}$$

In binary problems, Recall is also called True Positive Rate and corresponds to Sensitivity, whereas the True Negative Rate is also called Specificity. Recall and Precision per class can be computed for both the Positive and the Negative class. For imbalanced datasets, the F1-Score can be computed for each class^29^. The F1-Score for class *c* is defined as:

3 $$\begin{aligned} F{1- }Score_c = \frac{2\cdot Recall_c \cdot Precision_c}{Recall_c + Precision_c} \end{aligned}$$

and takes into account both Recall and Precision of the class. Thus, F1-Score takes into account the capability of the classification model to both correctly predict the samples of class *c* and to limit the amount of $FP_c$ samples.

## Results and discussion

The main results of this study are as follows:

- Trigeminal Blink Reflex (TBR) and Hand Blink Reflex (HBR) were recorded from a group of People with MS (PwMS) with Relapsing-Remitting form and from a healthy control group.
- Two AdaBoost classifiers were trained with TBR and HBR features each, for a binary classification task between PwMS and Controls.
- Both classifiers were able to identify PwMS with an accuracy over 70%.
- Most relevant features were highlighted for future investigations.

We focused on TBR, one of the most widely used reflex in clinical practice^30–35^, and on HBR to date never studied in MS. The ML algorithm trained on TBR or HBR data distinguished well between patients and healthy subjects, with results matching clinicians performing neurophysiological analysis^36^. Table 1 shows the performance achieved by the AdaBoost model in terms of average (with standard deviation) Recall, Precision, F1-Score per class over the five test folds, and overall Accuracy. It is worth noting how using HBR features provides sensitively better results than using TBR features. In addition to calculating average and standard deviation, in order to highlight performance differences we calculated an “absolute” performance value, meaning that performance is not calculated on 5 confusion matrices and then averaged, but directly on a single confusion matrix. Since there are no copies of the samples in the five test folds, we put the predictions made by the ML models over the 5 different folds together, and compared them to the original data at once, obtaining a single confusion matrix. These latter results confirmed better performance using HBR features.

As it can be observed, the standard deviation computed over the 5 folds used for tests is large for different metrics. This may be due to the limited size of the dataset; indeed, since each fold is composed of 6 samples, each misclassified sample reduces the fold accuracy by approximately 16.7%; similarly, recall and precision scores of each class are highly influenced by each error. It has been observed in previous studies that performing a random split of the data on small datasets may induce covariate shift and lead to a lower accuracy^37^. For this reason, we reported in the right panel of Table 1 also the performance achieved using a leave-one-out approach. Leave-one-out can be regarded as a special case of k-fold cross validation, in which each fold includes only one sample. In this way, each sample is taken apart as a test set once while the training and validation phases are performed on all the remaining samples, and, finally, a unique performance can be obtained for the whole dataset. The reported 90% accuracy when using only HBR features means that only 3 over 30 samples in the dataset are misclassified, one belonging to the PwMS class, and two to the control group. Conversely, 6 samples are misclassified when using only TBR features, resulting in an accuracy score of 80%.

**Table 1:** Average AdaBoost model performance over the 5 test folds, and total results using the leave-one-out approach, in terms of accuracy, recall, precision, and F1-Score per class, for different sets of features (only HBR features or TBR features). The “absolute” results refer to the scores computed over the total and single confusion matrix obtained by putting together the predictions over the 5 test folds.

Class	Metrics [%]	5-Fold Cross Validation				Leave-one-out	
		HBR		TBR		HBR	TBR
		$\mu +\sigma$	“absolute”	$\mu +\sigma$	“absolute”	μ (=“absolute”) + σ	μ (=“absolute”) + σ
	Accuracy	$86.7 \pm 7.5$	86.7	$73.3 \pm 19.0$	73.3	90.0 ± 30.5	80.0 ± 40.7
PwMS	Recall	$88.3 \pm 16.2$	88.2	$71.7 \pm 31.0$	70.6	94.1 ± 23.6	88.2 ± 32.3
	Precision	$91.0 \pm 12.4$	88.2	$82.7 \pm 16.7$	80.0	88.8 ± 31.5	78.9 ± 40.8
	F1-Score	$89.6 \pm 14.3$	88.2	$76.8 \pm 23.1$	75.0	91.4 ± 28.0	83.3 ± 37.3
Control	Recall	$83.3 \pm 23.6$	84.6	$76.7 \pm 22.4$	76.9	84.6 ± 36.1	69.2 ± 46.2
	Precision	$88.3 \pm 16.2$	84.6	$74.7 \pm 25.6$	66.7	91.7 ± 27.6	81.8 ± 38.6
	F1-Score	$85.8 \pm 19.9$	84.6	$75.7 \pm 16.5$	71.4	88.0 ± 32.5	75.0 ± 43.3

For all tests, we used AdaBoost with Decision Tree classifiers as weak learners. Such a method takes as input all the TBR or HBR features and, during the classification phase, it takes into account each feature based on its computed impurity-based importance^20,21^. During the Training phase, the AdaBoost classifier assigns to each of the *N* features an importance score $I_n$ ranging from 0 (the feature is not considered) to 1 (only that feature is considered) in such a way that $\sum _{n=1}^N I_n = 1$. The higher the $I_n$ score, the more important the feature. In other words, such a method performs a feature selection by not taking into account those features whose importance is computed as 0. Table 2 reports the features taken into account by the model and their average importance on the five model development folds. Features that are not reported in the table are always assigned an importance of 0 and, therefore, are never taken into consideration for the classification task. With regards to the HBR feature set, a total of 28 out of the 72 features are assigned an importance score greater than 0, but, for brevity purposes, we report only those features that are assigned an average importance greater than 0.05 on the five model development folds, and that are therefore more important for the classification task (a total of further 22 features are omitted).

**Table 2:** Feature importance scores. The features selected for the classification tasks using HBR or TBR data are sorted based on their importance. For brevity purposes, the HBR column only reports those features that achieve an average importance score greater than 0.05. These are the most important ones for the classification task.

HBR	TBR
Duration FAR DOWN eyeLEFT ipsilateralStimulation - 0.210	R2 LATENCY eyeLEFT controlateralStimulation - 0.846
Duration FAR UP eyeRIGHT controlateralStimulation - 0.170	R2 LATENCY eyeLEFT ipsilateralStimulation - 0.154
Duration FAR eyeRIGHT controlateralStimulation - 0.103	
Latency FAR eyeLEFT ipsilateralStimulation - 0.069	
Latency FAR DOWN eyeLEFT controlateralStimulation - 0.059	
Duration NEAR DOWN eyeRIGHT ipsilateralStimulation - 0.051	

**Figure 2:**
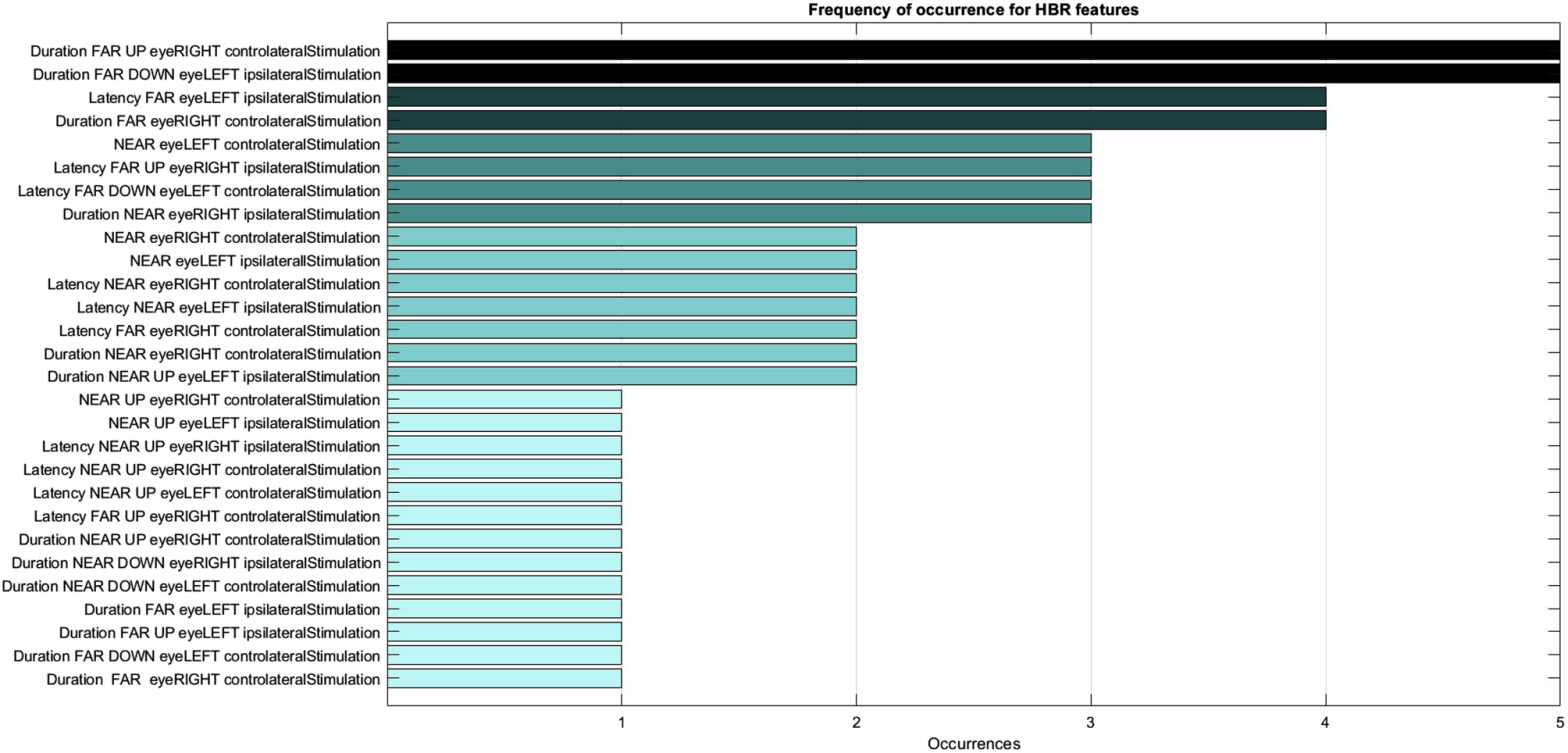
Frequency of occurrence of the HBR features over the five model development folds. Features which are always assigned an importance of 0 are not reported.

We present the complete frequency of occurrence of the HBR features in Fig. 2. Conversely, only two out of the 14 features of the TBR-only task present non-null importance. This may explain why considerably better performance is achieved when considering only HBR features rather than only TBR features.

In order to evaluate the statistical difference between the predictions of the models trained using the two sets of features, we performed a non-parametric Wilcoxon signed-rank test^38^. This is a sensible choice due to the small size of the data under consideration and since the accuracy scores do not follow a Gaussian distribution. The Wilcoxon test statistic computed from the comparison between the results of the two models is 0.0, whereas the computed p-value is 0.10. The test statistic result is due to the fact that the model based on HBR features achieves the same or higher accuracy on every fold of data then the one based on TBR features. The critical value of the Wilcoxon signed-rank test performed over 5 rank scores is 0.0^39^, thus, a statistically significant difference can be deduced between the prediction of these models. Moreover, due to the limited size of the dataset under consideration, we performed two additional tests for nonparametric data, namely Mann-Whitney U test^40^ and Fisher’s exact test^41^, in order to highlight the difference between the analyzed approaches. We performed these additional tests taking into account only the samples on which at least one of the two models provided a mistaken prediction, and investigated the difference between the predictions from the two models on these samples. This returned two sets of 10 predictions, only 2 of which were in common between the sets. This resulted in a p-value of 0.08 for the Mann-Whitney U test, and an odds ratio of 0.16 for the Fisher’s exact test. The latter result means that the probability to observe this or an even more imbalanced ratio by chance is just 16%. We can conclude that a statistically significant difference exists between the predictions produced using the two different sets of features.

The proposed AdaBoost model achieves better performance than the other models we tested on the dataset. All these models underwent the same validation procedure described for the AdaBoost in order to detect the optimal set of parameters for classification. Detailed performance of these models is reported in Table 3. It is worth noting how the AdaBoost trained with HBR features achieves the best performance among all the ML models. Interestingly, the k-NN is the only model to achieve a sensitively better performance when trained using the HBR features rather than TBR; conversely, the SVM, the RF, and the feedforward NN achieve better performance when trained with TBR features. In particular, the RF trained using TBR features is the second best performing ML model; however, its performance is worse than that achieved by the AdaBoost. The feedforward NN achieves the worst performance among all the models taken into consideration; this may be due to the relatively small dataset utilized that may not be sufficient to train a neural network model. Similarly to what happens for the AdaBoost model, large values of standard deviation are observed; for this reason, in order to provide a detailed comparison, we reported in Table 4 the performance of these models evaluated using a leave-one-out approach. Interestingly, the SVM and RF trained with TBR features provide the same performance for all metrics; differently, the RF trained with HBR features provides the same accuracy but with a difference in the other metrics. Also in this case, AdaBoost outperforms any other ML model.

**Table 3:** Average performance over the 5 test folds of the other ML models in terms of accuracy, recall, precision, and F1-Score per class, for different sets of features (only HBR features or only TBR features).

Class	Metrics [%]	k-NN		SVM		RF		NN	
		HBR	TBR	HBR	TBR	HBR	TBR	HBR	TBR
	Accuracy	$73.3\pm 19.0$	$56.7\pm 14.9$	$70.0\pm 21.7$	$73.3\pm 19.0$	$70.0\pm 21.7$	$83.3\pm 16.7$	$46.7\pm 32.0$	$50.0\pm 16.7$
PwMS	Recall	$95.0 \pm 11.1$	$70.0\pm 24.0$	$78.3\pm 21.7$	$75.0\pm 27.6$	$78.3\pm 21.7$	$86.7\pm 18.2$	$56.7\pm 39.7$	$66.7\pm 31.1$
	Precision	$72.3\pm 16.6$	$58.7\pm 17.7$	$73.7\pm 15.6$	$73.3\pm 18.1$	$73.3\pm 18.1$	$82.7\pm 16.7$	$48.7\pm 32.1$	$54.0\pm 13.0$
	F1-Score	$81.9\pm 14.3$	$63.8\pm 21.2$	$75.9\pm 17.3$	$74.1\pm 22.8$	$75.7\pm 19.3$	$84.6\pm 17.5$	$52.3\pm 35.7$	$59.7\pm 22.2$
Control	Recall	$40.0\pm 43.4$	$40.0\pm 9.1$	$56.7\pm 36.5$	$70.0\pm 18.2$	$60.0\pm 25.3$	$76.7\pm 22.4$	$30.0\pm 29.8$	$30.0\pm 18.3$
	Precision	$60.0\pm 54.8$	$56.7\pm 25.3$	$60.0\pm 43.5$	$73.3\pm 25.3$	$66.7\pm 31.1$	$86.7\pm 18.3$	$36.7\pm 41.5$	$53.3\pm 44.7$
	F1-Score	$50.4\pm 42.6$	$46.9\pm 16.3$	$58.2\pm 39.9$	$71.6\pm 21.6$	$63.1\pm 27.7$	$81.3\pm 20.2$	$33.0\pm 31.1$	$38.4\pm 29.4$

**Table 4:** Performance of the other ML models with a leave-one-out approach in terms of accuracy, recall, precision, and F1-Score per class, for different sets of features (only HBR features or only TBR features).

Class	Metrics [%]	k-NN		SVM		RF		NN	
		HBR	TBR	HBR	TBR	HBR	TBR	HBR	TBR
	Accuracy [%]	73.3 ± 44.2	63.3 ± 48.2	70.0 ± 45.8	80.0 ± 40.0	80.0 ± 40.0	80.0 ± 40.0	43.3 ± 49.6	40.0 ± 49.0
PwMS	Recall [%]	88.2 ± 32.3	70.6 ± 45.6	76.5 ± 42.4	82.3 ± 38.2	94.1 ± 23.6	82.3 ± 38.2	58.8 ± 49.2	35.3 ± 47.8
	Precision [%]	71.4 ± 45.2	66.7 ± 47.1	72.2 ± 44.8	82.3 ± 38.2	76.2 ± 42.6	82.3 ± 38.2	50.0 ± 50.0	46.2 ± 49.9
	F1-Score [%]	78.9 ± 40.8	68.6 ± 46.4	74.3 ± 43.7	82.3 ± 38.2	84.2 ± 36.5	82.3 ± 38.2	54.0 ± 49.8	40.0 ± 49.0
Control	Recall [%]	53.8 ± 49.9	53.8 ± 49.9	61.5 ± 48.7	76.9 ± 42.2	61.5 ± 48.7	76.9 ± 42.2	23.1 ± 42.1	46.2 ± 49.9
	Precision [%]	77.8 ± 41.6	58.3 ± 49.3	66.7 ± 47.1	76.9 ± 42.2	88.9 ± 31.4	76.9 ± 42.2	30.0 ± 45.8	35.3 ± 47.8
	F1-Score [%]	63.6 ± 48.1	56.0 ± 49.6	64.0 ± 48.0	76.9 ± 42.2	72.7 ± 44.6	76.9 ± 42.2	26.1 ± 43.9	40.0 ± 49.0

The classifier built on TBR features reproduces the results present in literature. Cabib and colleagues explored the characteristics of TBR in terms of response latency, response size and their lateral imbalance. With this analysis clinicians were able to correctly distinguish between healthy subjects and patients with altered BR responses. They were also able to find a greater number of lesions in MRI in those presenting altered TBR responses. On that occasion, they indicated a sensitivity (number of true positive) of 70% of patients correctly predicted referring to MRI data on a population of 20 patients^36^. Those results are comparable with the values obtained by our TBR classifier. Since, ML techniques seem to be a reliable tool to identify neurophysiological abnormalities in MS, providing an economical instrument to support clinicians in patient’s evaluation, even without MRI. Over time, in fact, the MRI technique has gained a major role in the diagnosis of MS: criteria for the diagnosis have changed based on new MRI criteria to allow an earlier diagnosis and reduce false-positive detection^42^. Furthermore, several efforts have been made to investigate the relationship between clinical outcomes and MRI, to find those elements prognostic for pathology severity^43,44^. However, in those early disease stages^45^, there is a weak correlation between lesions detected by MRI, symptoms and measures of disability such Expanded Disability Status Scale (EDSS)^46^. This mismatch between brain lesions and variability in clinical outcomes is called clinico-radiological paradox^46^. Researchers are now focusing on overcoming this issue by implementing MRI techniques focusing on micro-structure (such as diffusion tensor imaging) and on metabolic features (such as proton spectroscopy and perfusion)^47^. Further, the exploration of the involvement of Grey Matter and the atrophy of other CNS structures as spinal cord, thalamus and brainstem^48^ is promising in identifying the progression of the pathology. In view of this necessity, ML can prove to be a valuable alley to clinicians to apply in early diagnosis, in order to tailor therapy to each specific patient.

Despite TBR being already employed in clinical practice, our results showed that the classifier based on HBR features is even more precise than the other. HBR has never been explored in PwMS, and for this reason, its alteration has not been described yet. It has been proposed that brainstem alterations are present in 30–40% of PwMS, varying between different stages of pathology^6^. Different accuracy in classification could represent a different localization of lesions in our group of patients. Functional-anatomical differences in sensorimotor circuits that underlie TBR and HBR have been proposed: the former including the pontine reticular formation, the latter involving the mesencephalic reticular formation^4,49,50^. Furthermore, it has been proposed that distinct mechanisms underlie the two different spatial responses of HBR. The hand-far component, in which the stimulated hand is outside the DPPS, partially shares the same mechanism underlying the R2 component of TBR^14,24^. On the contrary, the brainstem interneurons mediating the hand-near component of HBR undergo a top-down regulation exerted by PZ and VIP areas, which have been suggested to encode and modulate the defensive behaviour within the DPPS. Such modulation is heterosegmentally specific for the brainstem interneurons mediating the HBR, which are thought to be different from those mediating the TBR^24^. If one focus on the feature relevance of the model (Fig. 2), in fact, could note that the two most used features by the algorithm are the FAR duration of the response during the voluntary movement session, meaning those regulated by a top-down modulation. However, our results suggest that HBR could be altered in patients with respect to healthy control. The ML tools are used to validate the efficiency of the new predictive method based on HBR. The ML classifiers performance suggests that the new method based on HBR is promising when applied in clinical practice as the TBR consolidated approach. HBR could also provide additional information about the integrity of brainstem circuits potentially favouring early diagnosis. Further investigation in MS field could be promising in exploring the relation between HBR alteration and brainstem functionality and needs further clinical research.

The main limitation of this study is the large amount of data necessary to train a machine learning model. Future developments may be directed towards the inclusion of a larger sample of patients to increase the amount of available data; this would allow to take into account the clinical variety of MS alterations. Furthermore, particular focus is necessary in the exploration of different forms of the pathology, especially for the MS types that could greatly benefit from early detection, such as CIS^51^.

## Data Availability

The datasets generated during and/or analyzed during the current study are available from the authors on reasonable request at monica.biggio@edu.unige.it.

## References

[1] Ysrraelit MC, Fiol MP, Gaitán MI, Correale J (2018). Quality of life assessment in multiple sclerosis: Different perception between patients and neurologists. Front. Neurol..

[2] Thompson A, Baranzini S, Geurts J, Hemmer B, Ciccarelli O (2018). Multiple sclerosis. Lancet.

[3] Nakashima I, Fujihara K, Okita N, Takase S, Itoyama Y (1999). Clinical and mri study of brain stem and cerebellar involvement in japanese patients with multiple sclerosis. J. Neurol., Neurosurg Psychiatry.

[4] Versace V (2020). Threat vs control: Potentiation of the trigeminal blink reflex by threat proximity is overruled by self-stimulation. Psychophysiology.

[5] Kimura J (1975). Electrically elicited blink reflex in diagnosis of multiple sclerosis. Review of 260 patients over a seven-year period. Brain.

[6] Dežmalj Grbelja L, Mikula I, Ćorić L, Stojić M, Demarin V (2021). The value of blink reflex in early diagnosis of multiple sclerosis. Acta Clin. Croat..

[7] Brooks JBB, Jardim MR, Papais-Alvarenga RM, Fragoso YD (2015). There is still a role for the blink reflex in the diagnosis and follow-up of multiple sclerosis. Clin. Neurophysiol..

[8] Magnano I (2014). Exploring brainstem function in multiple sclerosis by combining brainstem reflexes, evoked potentials, clinical and mri investigations. Clin. Neurophysiol..

[9] Cruccu, G. *et al.* Trigeminal neuralgia and pain related to multiple sclerosis. *PAIN* ® **143**, 186–191 (2009). 10.1016/j.pain.2008.12.026 19171430

[10] Cruccu G, Di Stefano G, Truini A (2020). Trigeminal neuralgia. N. Engl. J. Med..

[11] Bendtsen L (2019). European academy of neurology guideline on trigeminal neuralgia. Eur. J. Neurol..

[12] Mikropoulos EH, Papathanasiou AA, Hadjigeorgiou G, Tsironi E, Papadimitriou A (2010). Supratentorial multiple sclerosis lesions affect the blink reflex test. Open Neurol. J..

[13] Degirmenci E, Erdogan C, Bir LS (2013). Correlation between blink reflex abnormalities and magnetic resonance imaging findings in patients with multiple sclerosis. Acta Neurol. Belg..

[14] Sambo CF, Liang M, Cruccu G, Iannetti GD (2012). Defensive peripersonal space: The blink reflex evoked by hand stimulation is increased when the hand is near the face. J. Neurophysiol..

[15] Bisio A (2017). Dynamic shaping of the defensive peripersonal space through predictive motor mechanisms: when the “near” becomes “far”. J. Neurosci..

[16] Bufacchi RJ (2017). Approaching threatening stimuli cause an expansion of defensive peripersonal space. J. Neurophysiol..

[17] Biggio Monica, Bisio Ambra, Ruggeri Piero, Bove Marco (2019). Defensive peripersonal space is modified by a learnt protective posture. Sci. Rep..

[18] Seccia R (2021). Machine learning use for prognostic purposes in multiple sclerosis. Life.

[19] Brichetto G (2020). The hidden information in patient-reported outcomes and clinician-assessed outcomes: Multiple sclerosis as a proof of concept of a machine learning approach. Neurol. Sci..

[20] Freund Y, Schapire RE (1997). A decision-theoretic generalization of on-line learning and an application to boosting. J. Comput. Syst. Sci..

[21] Schapire, R. E. Explaining adaboost. 37–52 (2013).

[22] Polman CH (2011). Diagnostic criteria for multiple sclerosis: 2010 revisions to the mcdonald criteria. Ann. Neurol..

[23] Pfeiffer E (1975). A short portable mental status questionnaire for the assessment of organic brain deficit in elderly patients. J. Am. Geriatr. Soc..

[24] Mercante B (2020). Transcutaneous trigeminal nerve stimulation modulates the hand blink reflex. Sci. Rep..

[25] Pedregosa F (2011). Scikit-learn: Machine learning in Python. J. Mach. Learn. Res..

[26] Abdar M (2020). A new nested ensemble technique for automated diagnosis of breast cancer. Pattern Recogn. Lett..

[27] Zhong, Y., Chalise, P. & He, J. Nested cross-validation with ensemble feature selection and classification model for high-dimensional biological data. *Communications in Statistics-Simulation and Computation* 1–18 (2020).

[28] Ndiaye, E., Le, T., Fercoq, O., Salmon, J. & Takeuchi, I. Safe grid search with optimal complexity. In *International Conference on Machine Learning*, 4771–4780 (PMLR, 2019).

[29] D’Antoni F (2021). Artificial intelligence and computer vision in low back pain: A systematic review. Int. J. Environ. Res. Public Health.

[30] Cruccu G (2005). Brainstem reflex circuits revisited. Brain.

[31] Kimura J, Rodnitzky RL, Van Allen MW (1970). Electrodiagnostic study of trigeminal nerve: Orbicularis oculi reflex and masseter reflex in trigeminal neuralgia, paratrigeminal syndrome, and other lesions of the trigeminal nerve. Neurology.

[32] Kimura J, Lyon LW (1972). Orbicularis oculi reflex in the wallenberg syndrome: Alteration of the late reflex by lesions of the spinal tract and nucleus of the trigeminal nerve. J. Neurol., Neurosurg. Psychiatry.

[33] Kimura J (1983). Clinical uses of the electrically elicited blink reflex. Adv. Neurol..

[34] An electrophysiological and neuro-anatomical study of wallenberg’s syndrome (1978). Ongerboer de Visser, B. & Kuypers, H. Late blink reflex changes in lateral medullary lesions. Brain.

[35] Shahani B (1970). The human blink reflex. J. Neurol., Neurosurg. Psychiatry.

[36] Cabib C, Llufriu S, Martinez-Heras E, Saiz A, Valls-Solé J (2014). Abnormal control of orbicularis oculi reflex excitability in multiple sclerosis. PLoS ONE.

[37] Moreno-Torres JG, Sáez JA, Herrera F (2012). Study on the impact of partition-induced dataset shift on $$k$$-fold cross-validation. IEEE Transact. Neural Netw. Learn. Syst..

[38] Wilcoxon, F. Individual comparisons by ranking methods. In *Breakthroughs in Statistics*, 196–202 (Springer, 1992).

[39] sussex.ac.uk. Critical values of the wilcoxon signed ranks test, available at: http://users.sussex.ac.uk/~grahamh/rm1web/wilcoxonhandoout2011.pdf. Accessed: November 1, 2022.

[40] MacFarland, T. W. & Yates, J. M. Mann–Whitney U test. In *Introduction to Nonparametric Statistics for the Biological Sciences Using R*, 103–132 (Springer, 2016).

[41] Fisher, R. A. Statistical methods for research workers. In *Breakthroughs in Statistics*, 66–70 (Springer, 1992).

[42] Montalban X (2010). Mri criteria for ms in patients with clinically isolated syndromes. Neurology.

[43] Sormani MP (2009). Magnetic resonance imaging as a potential surrogate for relapses in multiple sclerosis: A meta-analytic approach. Ann. Neurol..

[44] Sormani M (2010). Surrogate endpoints for edss worsening in multiple sclerosis: A meta-analytic approach. Neurology.

[45] Roosendaal S (2009). Regional dti differences in multiple sclerosis patients. Neuroimage.

[46] Barkhof F (2002). The clinico-radiological paradox in multiple sclerosis revisited. Curr. Opin. Neurol..

[47] Chard, D. & Trip, S. A. Resolving the clinico-radiological paradox in multiple sclerosis. *F1000Research* **6** (2017). 10.12688/f1000research.11932.1 PMC5645703 29093810

[48] Filippi M (2020). Identifying progression in multiple sclerosis: New perspectives. Ann. Neurol..

[49] León L, Casanova-Molla J, Lauria G, Valls-Solé J (2011). The somatosensory blink reflex in upper and lower brainstem lesions. Muscle Nerve.

[50] Versace V (2021). Prepulse inhibition vs cognitive modulation of the hand-blink reflex. Sci. Rep..

[51] Tintore M (2010). Brainstem lesions in clinically isolated syndromes. Neurology.

